# A novel immunoinformatic approach for design and evaluation of heptavalent multiepitope foot-and-mouth disease virus vaccine

**DOI:** 10.1186/s12917-025-04509-1

**Published:** 2025-03-07

**Authors:** Mostafa R. Zaher, Mohamed H. El-Husseiny, Naglaa M. Hagag, Azza M. El-Amir, Mohamed E. El Zowalaty, Reham H. Tammam

**Affiliations:** 1 Genome Research Unit, Animal Health Research Institute, Agriculture Research Center (ARC), Giza, 12618 Egypt; 2 Department of Biotechnology, Faculty of Science, Cairo University, Giza, 12613 Egypt; 3 Reference Laboratory for Veterinary Quality Control on Poultry Production, Animal Health Research Institute, Agriculture Research Center (ARC), Giza, 12618 Egypt; 4 Department of Microbiology and Immunology, Faculty of Pharmacy, Ahram Canadian University, Giza, Egypt; 5 Department of Chemistry, Faculty of Science, Cairo University, Giza, 12613 Egypt

**Keywords:** Immunoinformatics, Multiepitope, Vaccine, FMDV, Molecular docking, Molecular dynamics

## Abstract

**Background:**

Foot-and-mouth disease virus (FMDV) vaccine development can be a laborious task due to the existence of various serotypes and lineages and its quasi-species nature. Immunoinformatics provide effective and promising avenue for the development of multiepitope vaccines against such complex pathogens. In this study, we developed an immunoinformatic pipeline to design a heptavalent multi-epitope vaccine targeting circulating FMDV isolates in Egypt.

**Result:**

B and T-cell epitopes were predicted and selected epitopes were proved to be non-allergenic, non-toxic, with high antigenicity, and able to induce interferon-gamma response. The epitopes were used to construct a vaccine by adding suitable linkers and adjuvant. Prediction, refinement, and validation of the final construct proved its stability and solubility, having a theoretical isoelectric point (PI) of 9.4 and a molecular weight of 75.49 kDa. The final construct was evaluated for its interaction with bovine toll-like receptor (TLR) 2 and 4 using molecular docking analysis and molecular dynamic simulation showed high binding affinity, especially toward TLR4. MM/GBSA energy calculation supported these findings, confirming favorable energetics of the interaction. Finally, the DNA sequence of the vaccine was cloned in pET-30a (+) for efficient expression in *Escherichia coli*.

**Conclusion:**

The inclusion of computational and immunoinformatic approaches will ensure cost-effectiveness and rapid design of FMDV vaccine, decrease wet lab experimentation, and aid the selection of novel FMDV vaccines. While the vaccine demonstrates promising in-silico results, experimental assessment of vaccine efficiency is required.

**Supplementary Information:**

The online version contains supplementary material available at 10.1186/s12917-025-04509-1.

## Background

Foot-and-mouth disease virus (FMDV) is one of the highly endemic viruses that can infect various cloven-hoofed species. FMDV causes Foot-and-mouth disease (FMD), a non-fatal disease in adult animals, but has a high mortality rate in younglings and causes significant animal abortions [1, 2]. Despite the low level of viral mortality in adult animals, there is a high proportion of infectivity between animals in proximity, due to the easy spread of the virus between them [3]. FMD was reported to cause 8–22 billion USD in economic losses each year in endemic countries [4].

FMDV consists of positive-sense single-stranded RNA with around ~ 7000 nucleotides (nt) open reading frame, that encodes for all the structural and non-structural proteins. Other regions for the single-stranded RNA are designated for the 5` and 3` untranslated regions that measure around 1300 and 90 nt in length, respectively [5, 6]. The viral open reading frame is translated upon entering the mammalian cell into a polyprotein that is cleaved into 15 mature proteins divided into structural (VP1, VP2, VP3, and VP4) -that assemble to form the viral capsid- and non-structural proteins (Lab, Lb, 2 A, 2B, 2 C, 3 A, 3B1, 3B2, 3B3, 3 C, and 3D) [7].

FMDV has seven different serotypes (A, O, Asia1, C, SAT1, SAT2, and SAT3) where SAT serotypes are localized mainly in Africa. This classification of FMDV was developed based on serological analysis [8] and supported by VP1 sequence analysis [9, 10]. RNA replication errors [11], genome recombination [12, 13], and selection pressure imposed by the host immune system [14] are responsible for the high sequence variability of the FMDV genome, sequentially caused the emergence of different serotype variants. Most sequence variability is present in the structural protein-coding sequence, with VP1 showing the lowest identity scores between different serotypes ranging from 50 to 70%. Whereas the most variabilities were found in the exposed parts of proteins (VP1-3) of the viral capsid, VP4 protein showed less variability within structural proteins [14].

The production of an effective FMDV vaccine that induces sterile and solid immunity has not been achieved despite the ongoing research effort during the last decades, resulting in intensive viral spread, especially in enzootic countries [15]. The lack of an effective vaccine can be reasoned for viral quasi-species and the emergence of new FMDV variants every year. The high variabilities among FMDV serotypes restrain the ability to reach a universal vaccine that provides reliable protection from the seven known viral serotypes [16]. Authorities resort to use vaccines consisting of different formulations of inactivated virus serotypes [17]. Current approaches for developing FMDV vaccine aim to produce multi-epitope vaccines (MEV) that provide a solid immune response with a low risk of infectivity.

The wide availability of different databases for most biological information, such as molecular biology and genetics, enabled the development of various computational tools for in-silico experimentation to be conducted before application in a laboratory environment [18, 19]. Improvement of computational power and introduction of Immunoinformatics traverse the paradigm of vaccine design to sequence-based techniques instead of traditional methodologies [20]. In addition to recombinant DNA technology, immunoinformatics helped in the preparation of new peptides with better antigenic and immunological characteristics increasing the efficiency of the designed vaccine [21, 22].

Immunoinformatics, with the aid of machine learning and artificial neural network models, have a great role in the selection of those peptides for different immunocytes with high prediction specificity and sensitivity [23–25]. In-silico prediction of effective epitopes can have a huge impact in decreasing wet lab experiments and in turn, increasing vaccine design efficiency [26]. Different statistical models were developed and validated to evaluate the designed peptides based on the genomic and amino-acid sequence variability [27–29].

**Fig. 1:**
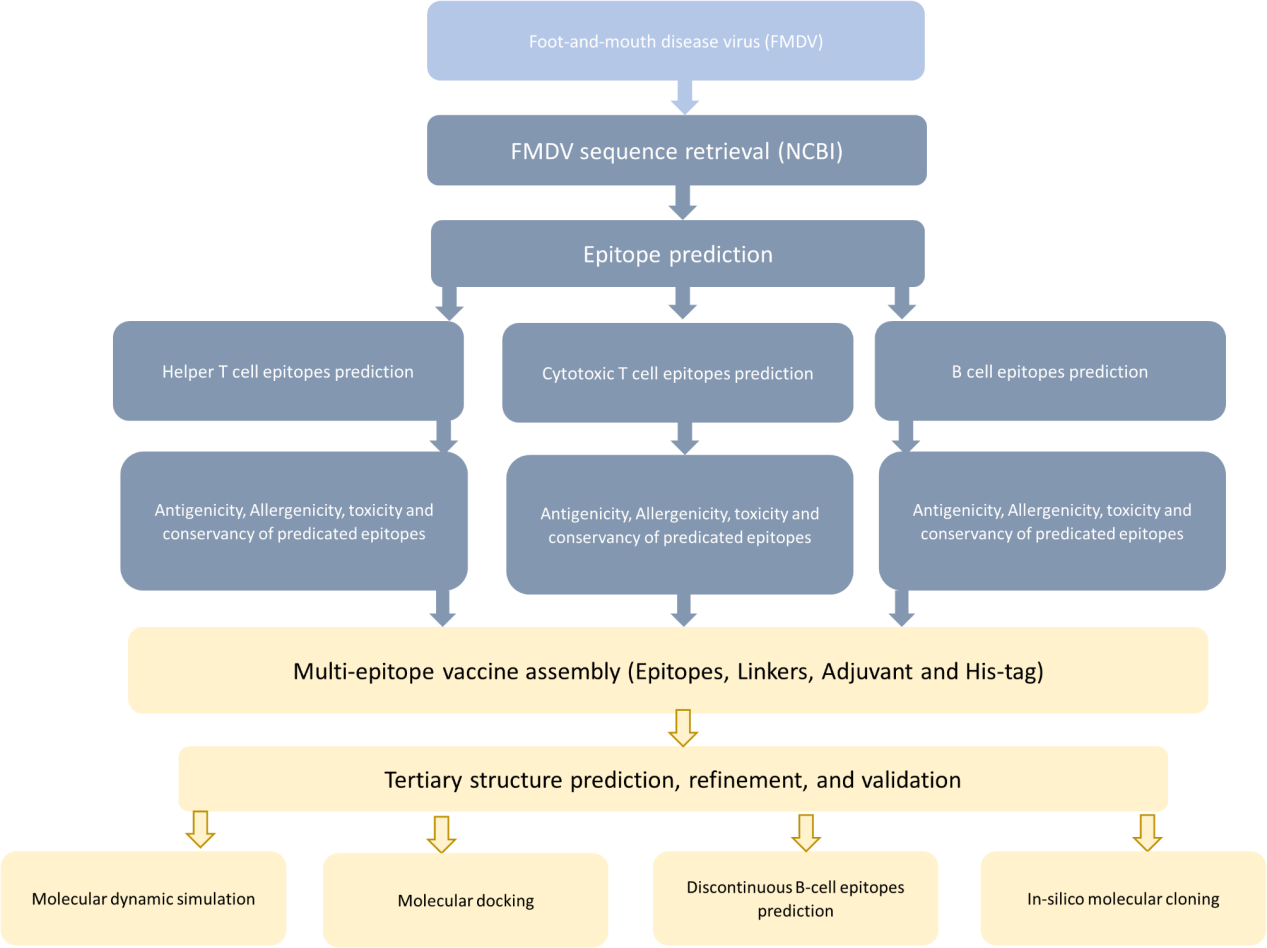
Illustration of the study workflow

In this study, we harnessed the power of immunoinformatics to develop and evaluate a new FMDV multiepitope-based vaccine for circulating serotypes in Egypt. Our workflow pipeline (Fig. 1) starts with the identification of efficient structural protein epitopes for B and T cells. Selected epitopes are connected through specific protein linkers to each other and to the adjuvant for the generation of MEV. The proposed pipeline provides a good pathway for the development of the FMDV vaccine industry and increases the knowledge of FMDV control.

## Methods

### Protein sequence retrieval

Complete FMDV proteomes of serotypes A, O and SAT2 were retrieved from the National Center of Biotechnology Information (NCBI) protein database in FASTA format (https://www.ncbi.nlm.nih.gov, accessed on 15 February 2022) (Table 1). All amino acid sequences were trimmed to include only the structural proteins (VP1, VP2, and VP3). The trimmed amino acid sequences were later used for epitope prediction.

**Table 1:** Protein sequences retrieved from the NCBI protein database

Serotype	Topotype	Lineage	Isolate	Accession no.	Length (amino acid)
O	East Africa 3 (EA-3)	-	ETH/9/2019	QNT13150	734
	Middle East- South Asia (ME-SA)	-	O/manisa/87	AAT01766	734
	Middle East- South Asia (ME-SA)	PanAsia-2	O/IRN/8/2005	AMR58284	734
A	Asia	Iran05	A/SIN/PAK/L693/2009	AEB02102	
	Africa	G-VII	A/UGA/13/66	AGO58325	
	Africa	GIV	A/SUD/3/77	AGO58316	
SAT 2	VII	Lib-12	EGY/1/2018	QNT13156	740
	VII	Ghb-12	EGY/9/2012	AFP33712	740

### Epitope prediction

#### Cytotoxic T lymphocytes (CTL)

Epitopes of the CD8 + T cell were predicted using major histocompatibility I (MHC-I) binding prediction tool, immune epitope database (IEDB) webserver, (http://tools.iedb.org/mhci/). The web tool depends on the (NetMHCpan EL 4.1) updated version, which has been trained in extended MHC-I binding affinity, and multi and single-allelic eluted ligands data from different public domains [30]. The tool’s machine learning algorithm was also updated from NNAlign to NNAlign_MA to include the multi-allelic MHC data [30, 31]. Available Bovine MHC-I alleles in the IEDB database (Table s1) were aligned against the query FMDV protein sequences, and data less than the percentile rank threshold (< 0.05) were selected.

#### Helper T lymphocytes (HTL)

NetMHCIIpan 2.1 server (https://services.healthtech.dtu.dk/service.php?NetMHCIIpan-2.1*)* was used to predict CD4 + T cell epitopes. The server uses artificial neural networks (ANN) that have been trained in more than 200,000 randomly selected peptides [32]. The server was selected because it includes Bovine leukocyte alleles (BoLA) of MHC-II (i.e., BoLA-DRB3) (Table s2). One of three parameters was used for the selection of epitopes that may have a strong binding affinity with selected MHC-II alleles. These parameters are binding affinity (IC50), percentile rank, and prediction score with thresholds of < 50 nM, < 0.5, and > 0.9, respectively.

#### B-cells

Linear B-cell epitopes were predicted using the ABCpred web tool (http://crdd.osdd.net/raghava/abcpred/). ABCpred uses ANN to predict B-cell epitopes depending on four metrics generated during the prediction process, precision, sensitivity, positive predictive value, and specificity. The prediction accuracy of the webtool model is 65.93% with the use of the cross-validation fold [33]. The FASTA sequences of all selected FMDV serotypes in the study were used to predict the B-cell epitopes, with a threshold of > 0.5 and peptide length of 16 mer.

### Allergenicity, toxicity, antigenicity, and conservancy analysis of epitopes

Selected epitopes were evaluated for their antigenicity using the Vaxijen v2.0 server (http://www.ddg-pharmfac.net/vaxijen/*)* by default score of > 0.4 for the antigenic epitope [34, 35]. Peptides that showed no antigenicity or value near the threshold were discarded.

Allergenicity and toxicity of the selected epitopes were identified using the AllerTop server (http://www.ddg-pharmfac.net/AllerTop/) [36] and ToxinPred server (http://crdd.osdd.net/raghava/toxinpred/) [37], respectively. Epitopes that were identified as allergenic or toxic were discarded.

High-level antigenic epitope conservancy is required to ensure that the vaccine can provide wider protection against different strains. Hence, the predicted epitopes were compared against 150 amino acid sequences for each serotype retrieved from the NCBI protein database using the BLASTp tool against the FMDV serotype P1 protein. The epitope conservancy analysis tool from the IEDB web server (http://tools.iedb.org/conservancy/) was used for the analysis of the epitope conservancy pattern with a threshold of 0.8 for sequence identity [38].

### Design of MEV

The MEV was designed by fusing B-cell and T-cell epitopes. These epitopes were highly conserved within the same serotype and showed high antigenicity, non-allergenicity, and non-toxicity. “KK”, “AAY” and “GPGPG” linkers were used to connect epitopes from B-cell, cytotoxic T-cell, and helper T-cells, respectively. Mycobacterium tuberculosis heparin-binding hemagglutinin (HBHA) was used as an adjuvant in the vaccine construct as it was proved to be an efficient TLR-4 agonist, enhancing immune activation. For proper separation and flexibility between the adjuvant and the multiepitope region, an “EAAAK” linker was used.

### MEV construct, TLR-4, and TLR-2 structure modeling, refinement, and validation

The tertiary structure of MEV was predicted using the Robetta server (Robetta server, https://robetta.bakerlab.org/*).* The Robetta server is a protein prediction server, its core is the Rosetta macromolecular modeling suite [39]. The RoseTTAFold option, a deep learning-based method, was chosen for the prediction of the MEV tertiary structure. The web server generated multiple models for the tertiary structure of the MEV. The bovine TLR-4 protein sequence (accession number NP_776623) and TLR-2 protein sequence (accession number ALL55248.1) were aligned against the SWISS-Model database [40] to build their tertiary structures using homology modeling. The web server ranked the model according to their highest accuracy scores.

The generated tertiary structures models were then refined and enhanced using GalaxyRefine (https://galaxy.seoklab.org/cgi-bin/submit.cgi?type=REFINE) web server [41, 42]. The residual geometry of the refined vaccine construction was analyzed and improved followed by the prediction of construction stereochemical quality using the PROCHECK web tool from the SAVES v6.0 server (https://saves.mbi.ucla.edu/) [43]. Data generated from the Ramachandran plot and ERRAT scores were used for the selection of the best tertiary model. The final structure of the selected model was tested for the presence of errors using the ProsA server (https://prosa.services.came.sbg.ac.at/prosa.php) [44]. The final optimized protein structure was evaluated for its physicochemical properties using ProtParam, Expasy webserver (https://web.expasy.org/protparam/).

### Discontinuous B-cell epitope prediction

Discontinuous B-cell epitopes of MEV 3D structure were predicted using the Ellipro tool in the IEDB webserver (http://tools.iedb.org/ellipro/). The Ellipro tool can predict both linear and discontinuous epitopes. It uses three main algorithms to predict the discontinuous epitopes. These algorithms work on identification of the epitopes by clustering protein residues based on certain values called Protrusion Index (PI), their spatial proximity, and giving them scores determined by the PI of their residues. The web tool was used with its default parameters with a minimum score of 0.5 and a maximum distance of 6 angstroms.

### Molecular docking of TLR-4 and TLR-2 with MEV

Protein-protein interaction between the MEV and both bovine TLR-4 and TLR-2 were predicted using Clustpro server (https://cluspro.org/login.php?redir=/models.php?job=1177853) [45]. The structures with interaction within the extracellular region of TLR-4 or TLR-2 were selected for further analysis of the docked complex. Binding affinities ($\:\varDelta\:G)$ and Equilibrium dissociation constant (K_d_) of selected models was determined using the PRODIGY webserver (https://wenmr.science.uu.nl/prodigy/) [46]. The interacting atoms were identified and visualized using the PDBsum server (https://www.ebi.ac.uk/thorntonsrv/databases/pdbsum/Generate.html) [47]. The final docked complexes were visualized using Discovery Studio 2023 (https://www.3ds.com/products/biovia/discovery-studio).

### Molecular dynamic simulation and energy minimization

The topology parameters of the proteins were generated using GROMACS 2024.2 with AMBER99SB force field [48]. Each protein complex was solvated in a cubic box with SPC water model [49] and neutralized by adding Cl^−^ counter ions. The neutralized systems underwent energy minimization using the steepest descent algorithm in 50,000 steps or Fmax < 400 kJ/mol. Subsequently, the minimized systems were equilibrated in two steps: first, NVT ensemble where the temperature was controlled at 300 K for 200 ps using the V-rescale thermostat, followed by NPT ensemble where the pressure was controlled by Parrinello-Rahman barostat algorithm for another 200 ps. Finally, the MD simulations were performed for 100 ns under NPT ensemble with time step of 2 fs. Long-range electrostatic interactions were calculated using Particle Mesh Ewald (PME) algorithm [50]. Hydrogen bond lengths were constrained using Linear Constraint Solver (LINCS) algorithm [51]. The trajectories from the production run were treated for removing the periodic boundary conditions (PBC) and then used in the analysis.

Following the MD simulation, the root mean square deviations (RMSD) from initial equilibrated positions of C-α atoms of each chain of TLRs was investigated to test the stability of respective proteins. The fluctuations in the side chain atoms of residue of the tested TLR protein and were analysed as root mean square fluctuation (RMSF) of C-α atoms of each protein in systems. The compactness of system and consequent stability was analysed in terms of radius of gyration (Rg) for each protein of the TLRs. The hydrogen bonds formed between vaccine chain and the TLR chains were analysed using appropriate index files of respective chains. the trajectories were analysed using the GROMACS tools *rms* for RMSD, *rmsf* for RMSF, *gyrate* for RG, *sasa* for SASA and *hbond* to calculate the number of hydrogen bonds.

The docked protein-vaccine complexes were imported into the **HawkDock** server V2 (http://cadd.zju.edu.cn/hawkdock/) for VD-MM/GBSA analysis [52]. The binding free energy of each complex was calculated, and key residues contributing to the interaction were identified through per-residue energy decomposition.

### MEV codon optimization and in-silico cloning

Reverse translation and codon optimization were generated using the Reverse translate tool, sequence manipulation suite (https://www.bioinformatics.org/sms2/rev_trans.html), and codon optimization tool, VectorBuilder (https://en.vectorbuilder.com/tool/codon-optimization.html), respectively. The generated cDNA sequence was optimized for expression in the *E. coli* K-12 strain. The DNA sequence was inserted into the pET-30a (+) vector using the SnapGene tool.

## Results

### Strains selection and epitope prediction

Protein sequences representing Egyptian circulating FMDV strains for O, A, and SAT2 serotypes were retrieved from the NCBI protein database. Whole genomes or full P1 sequences for all strains were obtained and in case, the full-length sequences were not found for a certain strain, the full sequence of the nearest strain was retrieved. For instance, the ETH/9/2019 genomic sequence was used as the nearest replacement for the circulated Egyptian East Africa 3 (EA-3) strain. Those sequences were later prepared and subjected to epitope prediction.

**Fig. 2:**
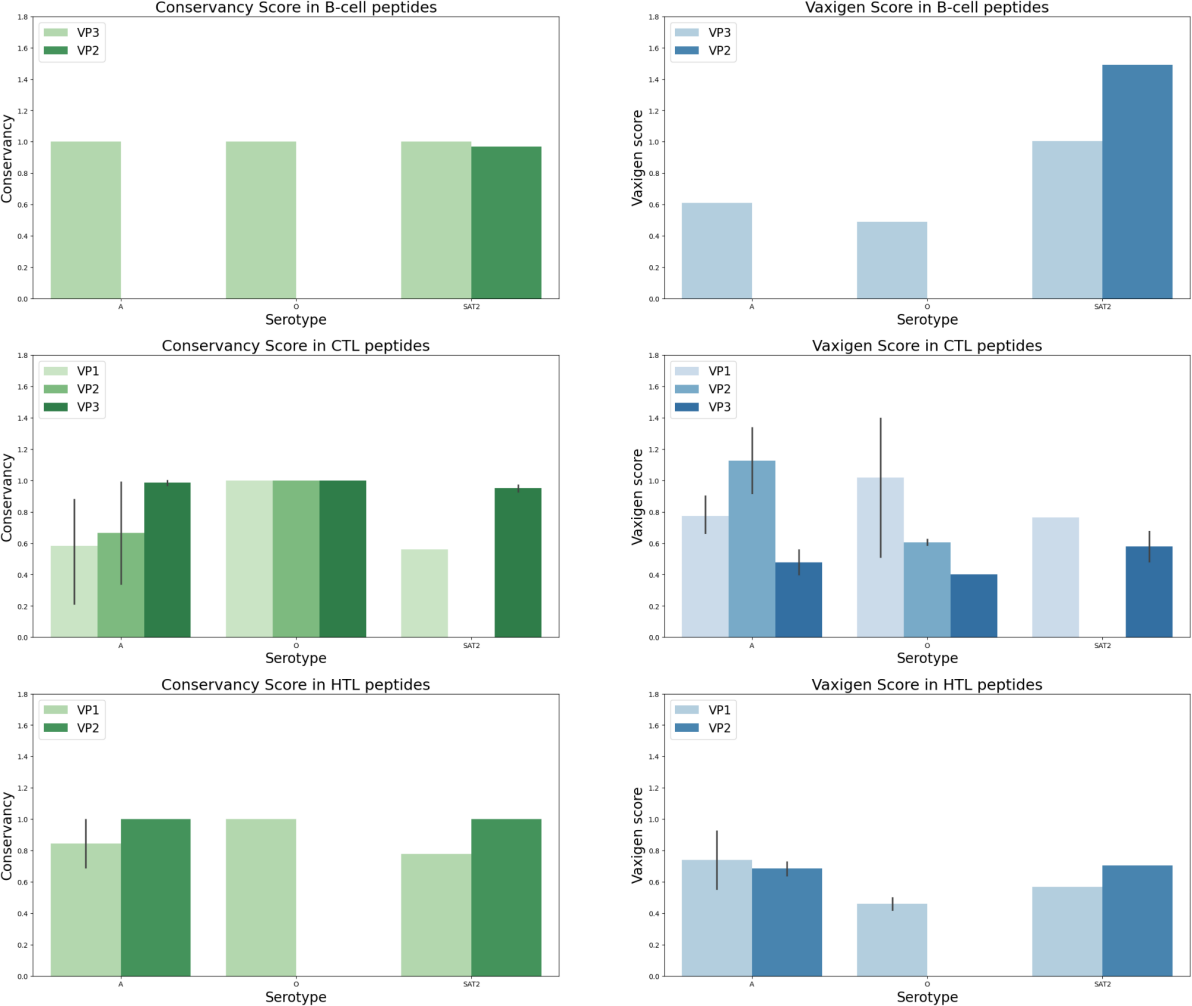
Conservancy and antigenicity analysis for the predicted surface antigen epitopes for (1) BCL, (2) CTL and (3) HTL

Predicted epitopes that showed high binding affinity to B- and T-cell alleles were considered for the vaccine design. epitope conservancy analysis that showed high conservancy within its serotype (> 80%) with high antigenicity were selected. (Fig. 2).

#### CTL predicted epitopes

T-cell epitopes were included in preventive vaccines to naturally mimic the immune response triggered by infection. T-cell epitopes help in the induction of durable cellular-mediated immunity. Epitopes with the highest binding scores and the lowest percentile rank (< 0.05) against 103 BoLA were selected as strong binders (Table 2). More than 62 epitopes were higher than 0.85 binding score, and lower than 0.05 in percentile rank. After antigenic and allergenic filtration, fifteen different alleles showed high binding affinity toward 16 different epitopes in all tested serotypes. Epitope starts at position 402 of P1 has been predicted in the three serotypes and has a binding affinity toward four different MHC-I alleles (BoLA-1:02301, BoLA-6:01301, BoLA-D18.4, and BoLA-HD6). Although the position of the epitope was conserved in all the serotypes, its amino acid sequence was different, particularly in the SAT2 serotype. On the other hand, epitope “DVAEACPTL” was predicted to have a high affinity toward MHC-I from SAT2 VP3 protein. Although it was only predicted within the VP3 protein sequence, the conservancy analysis of this epitope showed to be conserved in the three serotypes.

**Table 2:** List of CTL (MHC-I) predicted peptides including conservancy and antigenicity score

seq_num	Start	Allele	Peptide	Score	Percentile_rank	AllerTop	Toxinpred	Vaxigen SQ	Cons-O	Cons-A	Cons-SAT2
A	VP1	BoLA-3:01101	KSLGPTHVI	0.90301	0.01	NON-ALLERGEN	Non-Toxin	0.7081	0.00%	1.03%	0.00%
		BoLA-2:00801	VVKHQGNLTW	0.85837	0.02	NON-ALLERGEN	Non-Toxin	0.9652	99.08%	94.33%	0.00%
		BoLA-2:01201	TSNPTAYHK	0.88209	0.03	NON-ALLERGEN	Non-Toxin	0.652	94.47%	82.47%	0.00%
		BoLA-T2a		0.88209	0.03	NON-ALLERGEN	Non-Toxin	0.652	94.47%	82.47%	0.00%
		BoLA-1:02301	AQLPASFNY	0.86820	0.02	NON-ALLERGEN	Non-Toxin	0.769	0.00%	56.19%	0.00%
		BoLA-D18.4		0.86820	0.02	NON-ALLERGEN	Non-Toxin	0.769	0.00%	56.19%	0.00%
	VP2	BoLA-1:02301	YKNHKPWTL	0.86592	0.02	NON-ALLERGEN	Non-Toxin	0.9194	98.62%	99.48%	0.00%
		BoLA-D18.4		0.86592	0.02	NON-ALLERGEN	Non-Toxin	0.9194	98.62%	99.48%	0.00%
		BoLA-2:01201	SSIGASQIK	0.86034	0.05	NON-ALLERGEN	Non-Toxin	1.3363	0.00%	33.51%	0.00%
		BoLA-T2a		0.86034	0.05	NON-ALLERGEN	Non-Toxin	1.3363	0.00%	33.51%	0.00%
	VP3	BoLA-1:02301	AQYSGTINL	0.94326	0.01	NON-ALLERGEN	Non-Toxin	0.5566	100.00%	100.00%	0.00%
		BoLA-6:01301		0.96461	0.02	NON-ALLERGEN	Non-Toxin	0.5566	100.00%	100.00%	0.00%
		BoLA-D18.4		0.94326	0.01	NON-ALLERGEN	Non-Toxin	0.5566	100.00%	100.00%	0.00%
		BoLA-HD6		0.96461	0.02	NON-ALLERGEN	Non-Toxin	0.5566	100.00%	100.00%	0.00%
		BoLA-1:02301	TQYSGTINL	0.91057	0.02	NON-ALLERGEN	Non-Toxin	0.4008	100.00%	97.42%	0.00%
		BoLA-6:01301		0.89276	0.05	NON-ALLERGEN	Non-Toxin	0.4008	100.00%	97.42%	0.00%
		BoLA-D18.4		0.91057	0.02	NON-ALLERGEN	Non-Toxin	0.4008	100.00%	97.42%	0.00%
		BoLA-HD6		0.89276	0.05	NON-ALLERGEN	Non-Toxin	0.4008	100.00%	97.42%	0.00%
		BoLA-T2c		0.91577	0.05	NON-ALLERGEN	Non-Toxin	0.4008	100.00%	97.42%	0.00%
O	VP1	BoLA-6:01402	GESADPVTA	0.86797	0.04	NON-ALLERGEN	Non-Toxin	0.5132	100.00%	100.00%	0.00%
		BoLA-2:00801	AVKHEGNLTW	0.93548	0.01	NON-ALLERGEN	Non-Toxin	1.1427	100.00%	50.52%	0.00%
		BoLA-1:02301	IKATRVTEL	0.85480	0.03	NON-ALLERGEN	Non-Toxin	1.3971	99.54%	0.00%	0.00%
		BoLA-D18.4		0.85480	0.03	NON-ALLERGEN	Non-Toxin	1.3971	99.54%	0.00%	0.00%
	VP2	BoLA-6:01301	SIQKRELYQL	0.95782	0.02	NON-ALLERGEN	Non-Toxin	0.5893	100.00%	0.00%	0.00%
		BoLA-HD6		0.95782	0.02	NON-ALLERGEN	Non-Toxin	0.5893	100.00%	0.00%	0.00%
		BoLA-6:01301	IQKRELYQLTL	0.94439	0.03	NON-ALLERGEN	Non-Toxin	0.6233	100.00%	0.00%	0.00%
		BoLA-HD6		0.94439	0.03	NON-ALLERGEN	Non-Toxin	0.6233	100.00%	0.00%	0.00%
	VP3	BoLA-1:02301	TQYSGTINL	0.91057	0.02	NON-ALLERGEN	Non-Toxin	0.4008	100.00%	97.42%	0.00%
		BoLA-6:01301		0.89276	0.05	NON-ALLERGEN	Non-Toxin	0.4008	100.00%	97.42%	0.00%
		BoLA-D18.4		0.91057	0.02	NON-ALLERGEN	Non-Toxin	0.4008	100.00%	97.42%	0.00%
		BoLA-HD6		0.89276	0.05	NON-ALLERGEN	Non-Toxin	0.4008	100.00%	97.42%	0.00%
		BoLA-T2c		0.91577	0.05	NON-ALLERGEN	Non-Toxin	0.4008	100.00%	97.42%	0.00%
SAT2	VP1	BoLA-2:01801	IPFTAPHRL	0.94194	0.01	NON-ALLERGEN	Non-Toxin	0.7639	0.00%	0.00%	56.44%
		BoLA-2:01802		0.94194	0.01	NON-ALLERGEN	Non-Toxin	0.7639	0.00%	0.00%	56.44%
	VP3	BoLA-T2c	DVAEACPTL	0.93081	0.04	NON-ALLERGEN	Non-Toxin	0.6750	100.00%	100.00%	93.07%
		BoLA-1:00902	TQYSGSLNY	0.92714	0.01	NON-ALLERGEN	Non-Toxin	0.4857	0.00%	0.00%	96.53%
		BoLA-1:02301		0.94839	0.01	NON-ALLERGEN	Non-Toxin	0.4857	0.00%	0.00%	96.53%
		BoLA-2:06201		0.87324	0.01	NON-ALLERGEN	Non-Toxin	0.4857	0.00%	0.00%	96.53%
		BoLA-D18.4		0.94839	0.01	NON-ALLERGEN	Non-Toxin	0.4857	0.00%	0.00%	96.53%
		BoLA-T5		0.92714	0.01	NON-ALLERGEN	Non-Toxin	0.4857	0.00%	0.00%	96.53%

#### HTL predicted epitopes

HTL epitopes are essential for the generation of both humoral and cell-mediated immunity. These epitopes induce CD4 + responses that help in the formation of CD8 + T-cell memory and activation of antibody production by B-cells [53, 54]. We selected epitopes with the highest prediction score, the lowest percentile rank, and a binding affinity of less than 50 nM. The final selected epitopes were found to be mostly in VP1 protein (11 epitopes) and only three epitopes were found in VP2 protein (Table 3). Serotype A and SAT-2 showed epitopes within the same location of VP2 with different amino acid sequences, these epitopes had an affinity toward the same MHC-II alleles. Interestingly, the epitope sequences from the A serotype showed conservancy in O serotype isolates.

**Table 3:** List of HTL (MHC-II) predicted peptides including conservancy and antigenicity score

Serotype	Protein	MHC allele	Peptide	1-log15k(aff)	Affinity (nM)	%Rank	AllerTOP	Toxinpred	Vaxigen SQ	Cons-O	Cons-A	Cons-SAT2	IFNepitope
A	VP2	BoLA-DRB3*2705	WTLVVMVVSPLTTSS	0.837	4.77	0.5	NON-ALLERGEN	Non-Toxin	0.6423	8.92%	100.00%	0.00%	Negative
		BoLA-DRB3*2705	WTLVVMVVSPLTVST	0.838	4.74	0.5	NON-ALLERGEN	Non-Toxin	0.7264	96.82%	100.00%	0.00%	Negative
	VP1	BoLA-DRB3*6201	DRFVKISSLSPTHVI	0.831	5.07	0.5	NON-ALLERGEN	Non-Toxin	0.6932	0.00%	61.44%	0.00%	Positive
		BoLA-DRB3*1103		0.802	6.71	0.4	NON-ALLERGEN	Non-Toxin	0.6932	0.00%	61.44%	0.00%	Positive
		BoLA-DRB3*0901		0.8	6.82	0.2	NON-ALLERGEN	Non-Toxin	0.6932	0.00%	61.44%	0.00%	Positive
		BoLA-DRB3*0701		0.759	10.19	0.1	NON-ALLERGEN	Non-Toxin	0.6932	0.00%	61.44%	0.00%	Positive
		BoLA-DRB3*1301	RFVKISSLSPTHVID	0.845	4.45	0.4	NON-ALLERGEN	Non-Toxin	0.9474	0.00%	61.44%	0.00%	Negative
		BoLA-DRB3*6201		0.824	5.45	0.5	NON-ALLERGEN	Non-Toxin	0.9474	0.00%	61.44%	0.00%	Negative
		BoLA-DRB3*6101	NIHELLVRMKRAELY	0.942	1.74	0.2	NON-ALLERGEN	Non-Toxin	0.5358	0.00%	99.35%	0.00%	Positive
		BoLA-DRB3*0101		0.893	2.8	0.2	NON-ALLERGEN	Non-Toxin	0.5358	0.00%	99.35%	0.00%	Positive
		BoLA-DRB3*6402		0.821	5.61	0.4	NON-ALLERGEN	Non-Toxin	0.5358	0.00%	99.35%	0.00%	Positive
		BoLA-DRB3*6401		0.814	5.96	0.5	NON-ALLERGEN	Non-Toxin	0.5358	0.00%	99.35%	0.00%	Positive
		BoLA-DRB3*6101	TIHELLVRMKRAELY	0.944	1.72	0.2	NON-ALLERGEN	Non-Toxin	0.4825	0.00%	100.00%	0.00%	Positive
		BoLA-DRB3*0101		0.893	2.8	0.2	NON-ALLERGEN	Non-Toxin	0.4825	0.00%	100.00%	0.00%	Positive
		BoLA-DRB3*6402		0.823	5.51	0.4	NON-ALLERGEN	Non-Toxin	0.4825	0.00%	100.00%	0.00%	Positive
		BoLA-DRB3*6401		0.816	5.88	0.5	NON-ALLERGEN	Non-Toxin	0.4825	0.00%	100.00%	0.00%	Positive
		BoLA-DRB3*1103	HELLVRMKRAELYCP	0.801	6.75	0.4	NON-ALLERGEN	Non-Toxin	1.0335	100.00%	100.00%	0.00%	Positive
O	VP1	BoLA-DRB3*0101	DLQVLAQKAARALPT	0.868	3.56	0.5	NON-ALLERGEN	Non-Toxin	0.4978	100.00%	0.00%	0.00%	Negative
		BoLA-DRB3*1103		0.857	3.95	0.05	NON-ALLERGEN	Non-Toxin	0.4978	100.00%	0.00%	0.00%	Negative
		BoLA-DRB3*6401		0.848	4.32	0.15	NON-ALLERGEN	Non-Toxin	0.4978	100.00%	0.00%	0.00%	Negative
		BoLA-DRB3*1103	DLQVLAQKAARTLPT	0.804	6.56	0.4	NON-ALLERGEN	Non-Toxin	0.4232	100.00%	0.00%	0.00%	Positive
SAT2	VP2	BoLA-DRB3*2705	WSLVVMVLTPLTTEA	0.857	3.95	0.3	NON-ALLERGEN	Non-Toxin	0.7055	0.00%	0.00%	100.00%	Negative
	VP1	BoLA-DRB3*6201	ATAIRGDRAALAAKY	0.829	5.18	0.5	NON-ALLERGEN	Non-Toxin	0.5677	0.00%	0.00%	77.67%	Positive
		BoLA-DRB3*6402		0.821	5.58	0.4	NON-ALLERGEN	Non-Toxin	0.5677	0.00%	0.00%	77.67%	Positive
		BoLA-DRB3*1903		0.809	6.28	0.1	NON-ALLERGEN	Non-Toxin	0.5677	0.00%	0.00%	77.67%	Positive
		BoLA-DRB3*5901		0.795	7.17	0.2	NON-ALLERGEN	Non-Toxin	0.5677	0.00%	0.00%	77.67%	Positive
		BoLA-DRB3*1902		0.789	7.64	0.05	NON-ALLERGEN	Non-Toxin	0.5677	0.00%	0.00%	77.67%	Positive
		BoLA-DRB3*0501		0.788	7.65	0.05	NON-ALLERGEN	Non-Toxin	0.5677	0.00%	0.00%	77.67%	Positive
		BoLA-DRB3*0503		0.779	8.41	0.05	NON-ALLERGEN	Non-Toxin	0.5677	0.00%	0.00%	77.67%	Positive
		BoLA-DRB3*4001		0.775	8.67	0.5	NON-ALLERGEN	Non-Toxin	0.5677	0.00%	0.00%	77.67%	Positive
		BoLA-DRB3*0901		0.762	9.82	0.4	NON-ALLERGEN	Non-Toxin	0.5677	0.00%	0.00%	77.67%	Positive

#### B-cell predicted epitopes

B-cell epitopes were predicted using the ABCpred server where the algorithm sorts the predicted peptides according to their score in different ranks. Epitopes were filtered by selecting highly antigenic and non-toxic epitopes. Moreover, B-cell epitopes were tested for the presence of signal peptides. After filtration, only four epitopes showed promising B-cell epitopes from VP3 and VP2 proteins (Table 4). One peptide from SAT2 VP3 protein showed conservancy with the three serotypes.

**Table 4:** BCL-predicted epitopes for FMDV serotypes including conservancy and antigenicity scores

Serotype	Protein	Peptide	Score	Allertop	Toxipred	Vaxigen SQ	Cons-O	Cons-A	Cons-SAT2	signalP
A	VP3	GWVCVYQITHGKAEND	0.9	NON-ALLERGEN	Non-toxin	0.6100	2.55%	100.00%	0.00%	OTHER
O	VP3	AGLAQYYTQYSGTINL	0.88	NON-ALLERGEN	Non-toxin	0.4885	100.00%	99.35%	0.97%	OTHER
SAT2	VP2	LCSLKAREEFQLTLYP	0.87	NON-ALLERGEN	Non-toxin	1.4923	0.00%	0.00%	97.09%	OTHER
SAT2	VP3	DGYGGFQNTDPKTADP	0.87	NON-ALLERGEN	Non-toxin	1.0055	100.00%	99.35%	100.00%	OTHER

### MEV structure modeling, refinement, and validation

Vaccine construction involved 17 MHC-I epitopes, 11 MHC-II epitopes, and four B-cell epitopes. Those epitopes have been separated by AAY, GPGPG, and KK linkers, respectively. After the inclusion of HBHA adjuvant separated from B-cell epitopes with EAAAK linker and 6X his-tag, the final multiepitope protein length was 704 amino acids. The molecular weight of MEV was 75.49 KDa, theoretical PI 9.4, and the extension coefficient at 280 nm in water was 95,370 M^− 1^ cm^− 1^. The protein was predicted to be stable with an instability index of 27.5. The aliphatic index was 82.12 and the grand average of hydropathicity (GRAVY) was − 0.292, indicating that the protein is hydrophilic and soluble in water.

**Fig. 3:**
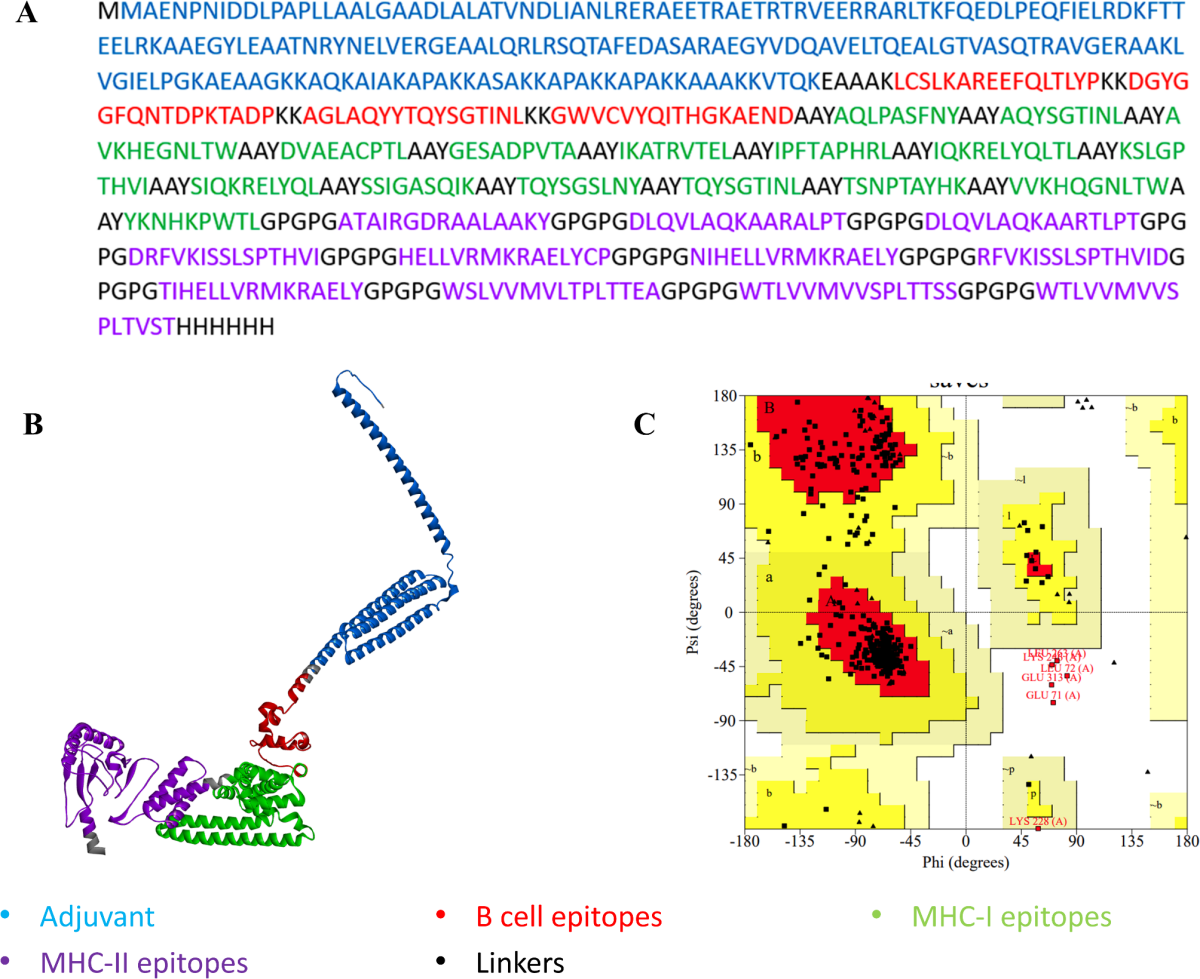
Modeled and refined structure of MEV. (**A**) amino acid sequence of MEV. (**B**) the tertiary structure of MEV. (**C**) Ramachandran plot analysis of predicted MEV structure

The tertiary structure of the protein was generated using the Robetta server. The generated models were further refined using the GalaxyRefine webserver. The validation scores of both original and refined models were used for the best model selection. The final selected model had an ERRAT score of 92.7, and its Ramachandran plot showed 92.9% residues within most favored regions, 6.1% residues in allowed regions, 0.2% residues in generously allowed regions, and 0.8% residues in disallowed regions. The final selected model Vaxigen score was 0.527, proving that the MEV is antigenic. The MEV was non-allergenic, non-toxic, and had no signal peptide nor transmembrane helices, so no specific protein localization nor difficulties could be faced during protein production (Fig. 3).

### Discontinuous B-cell epitopes prediction

**Fig. 4:**
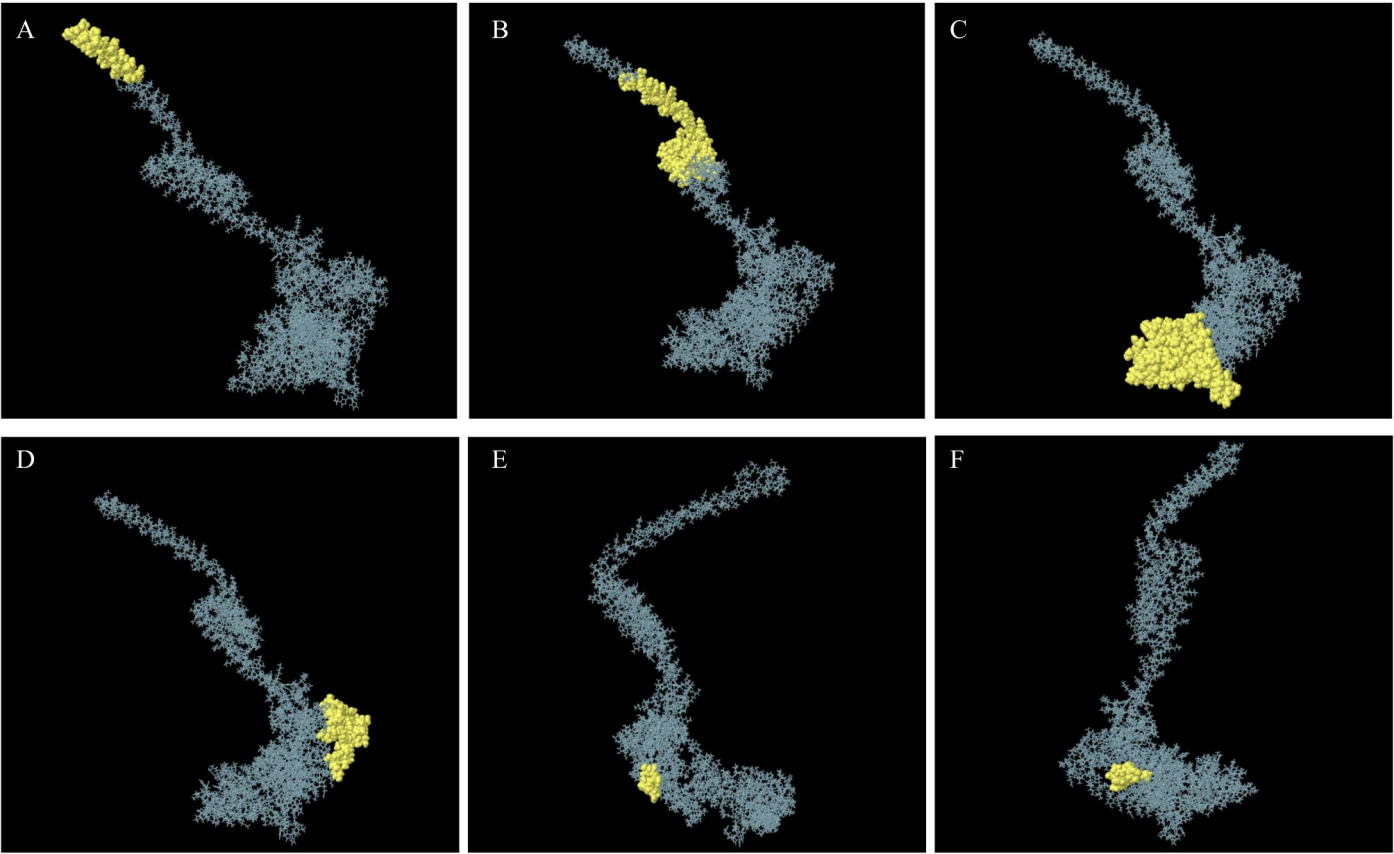
Discontinuous B-cell epitope prediction. Results showing epitope 1 to epitope 6 (**A**–**F**). The protein backbone is represented in blue and the yellow-highlighted residues represent the predicted discontinuous B-cell epitopes

A total of 359 residues were predicted to be involved in discontinuous B-cell epitopes. The length of predicted epitopes ranged from 9 to 147 residues with 37 residues having the highest binding score of 0.972 (Table 5) (Fig. 4). HBHA domain showed the highest epitope score and lowest epitope score located within MHCI epitopes.

**Table 5:** Discontinuous B-cell epitope prediction results showing involved residues within each epitope with its score

No.	Residues	Number of residues	Score
1	A: M2, A: A3, A: E4, A: N5, A: P6, A: N7, A: I8, A: D9, A: D10, A: L11, A: P12, A: A13, A: P14, A: L15, A: L16, A: A17, A: A18, A: L19, A: G20, A: A21, A: A22, A: D23, A: L24, A: A25, A: L26, A: A27, A: T28, A: V29, A: N30, A: D31, A: L32, A: I33, A: A34, A: N35, A: L36, A: E38, A: R39	37	0.972
2	A: R37, A: A40, A: E41, A: E42, A: T43, A: R44, A: A45, A: E46, A: T47, A: R48, A: T49, A: R50, A: V51, A: E52, A: E53, A: R54, A: R55, A: A56, A: R57, A: L58, A: T59, A: K60, A: F61, A: Q62, A: E63, A: D64, A: L65, A: P66, A: E67, A: Q68, A: F69, A: I70, A: E71, A: L72, A: R73, A: D74, A: K75, A: F76, A: T77, A: T78, A: E79, A: E80, A: L81, A: R82, A: K83, A: A84, A: A85, A: E86, A: G87, A: Y88, A: L89, A: E90, A: A91, A: A92, A: T93, A: N94, A: R95, A: Y96, A: N97, A: L133, A: T134, A: Q135, A: E136, A: A137, A: L138, A: G139, A: T140, A: V141, A: A142, A: S143, A: Q144, A: T145, A: R146, A: A147, A: V148, A: G149, A: E150, A: R151, A: A152, A: A153, A: K154, A: L155, A: V156, A: G157, A: I158, A: E159, A: L160, A: P161, A: G162, A: K163, A: A164, A: E165, A: A166, A: A167, A: G168, A: K169, A: K170, A: A171, A: Q172, A: K173, A: A174, A: I175, A: A176, A: K177, A: A178, A: P179, A: A180, A: K181, A: K182, A: S184	110	0.783
3	A: P542, A: T555, A: G559, A: P560, A: G561, A: P562, A: G563, A: E565, A: L566, A: L567, A: V568, A: R569, A: M570, A: K571, A: R572, A: A573, A: E574, A: L575, A: Y576, A: C577, A: P578, A: G579, A: P580, A: G581, A: P582, A: G583, A: N584, A: I585, A: H586, A: E587, A: L588, A: L589, A: V590, A: R591, A: M592, A: K593, A: R594, A: A595, A: E596, A: L597, A: Y598, A: G599, A: P600, A: G601, A: P602, A: G603, A: R604, A: F605, A: V606, A: K607, A: I608, A: S609, A: S610, A: L611, A: S612, A: P613, A: T614, A: H615, A: V616, A: I617, A: D618, A: G619, A: P620, A: G621, A: P622, A: G623, A: T624, A: I625, A: H626, A: E627, A: L628, A: L629, A: V630, A: R631, A: M632, A: K633, A: R634, A: A635, A: E636, A: L637, A: Y638, A: G639, A: P640, A: G641, A: P642, A: G643, A: W644, A: S645, A: L646, A: V647, A: V648, A: M649, A: V650, A: L651, A: T652, A: P653, A: L654, A: T655, A: T656, A: E657, A: A658, A: G659, A: P660, A: G661, A: P662, A: G663, A: W664, A: T665, A: L666, A: V667, A: V668, A: M669, A: V670, A: V671, A: S672, A: P673, A: L674, A: T675, A: T676, A: S677, A: S678, A: G679, A: P680, A: G681, A: P682, A: G683, A: W684, A: T685, A: L686, A: V687, A: V688, A: M689, A: V690, A: V691, A: S692, A: P693, A: L694, A: T695, A: V696, A: S697, A: T698, A: H699, A: H700, A: H701, A: H702, A: H703, A: H704	147	0.688
4	A: A407, A: Y408, A: S409, A: S410, A: I411, A: G412, A: A413, A: S414, A: Q415, A: I416, A: K417, A: A418, A: A419, A: Y420, A: T421, A: Q422, A: Y423, A: S424, A: G425, A: S426, A: L427, A: N428, A: Y429, A: A430, A: A431, A: Y432, A: T433, A: Q434, A: Y435, A: S436, A: T438, A: N447, A: T449, A: H452, A: K453, A: A454, A: A455, A: Y456, A: V457, A: V458, A: K459, A: H460, A: Q461, A: G462, A: N463, A: L464	46	0.666
5	A: A307, A: A309, A: V310, A: K311, A: H312, A: E313, A: G314, A: N315, A: L316	9	0.568
6	A: E353, A: A355, A: A356, A: Y357, A: I358, A: P359, A: F360, A: T361, A: P363, A: H364	10	0.54

### TLR-4 and TLR-2 structural prediction

Bovine TLR-4 and TLR-2 sequences were used to predict their tertiary structure using homology modeling by SWISS-Model from the Expasy webserver. SWISS-Model generated different tertiary structures based on sequence alignment with PDB and AlphaFold database. TLR-4 tertiary structure showed 84% similarity with the TLR-4 *Neophocaena asiaeorientalis* model from the AlphaFold database. TLR-2 highest model showed 95% similarity with *Capra ibex* TLR-2. Selected models were refined using GalaxyRefine and RMSD values were 0.338 and 0.3 for TLR-4 and TLR-2, respectively. Ramachandran plot and quality score were generated using ERRAT and PROCHECK from the SAVES V6.0 website. The overall quality factor for TLR-4 and TLR-2 were 88.3 and 91.6, respectively. TLR-4 Ramachandran plot showed that the refined model is reliable with 89.4% atoms in the favored regions, 10.1% in the allowed regions, and 0.4% in disallowed regions. TLR-2 had 90.3% of the molecular atoms in the favored regions, 9.5% in the allowed regions, and 0.1% in the disallowed regions.

### Molecular docking analysis

**Fig. 5:**
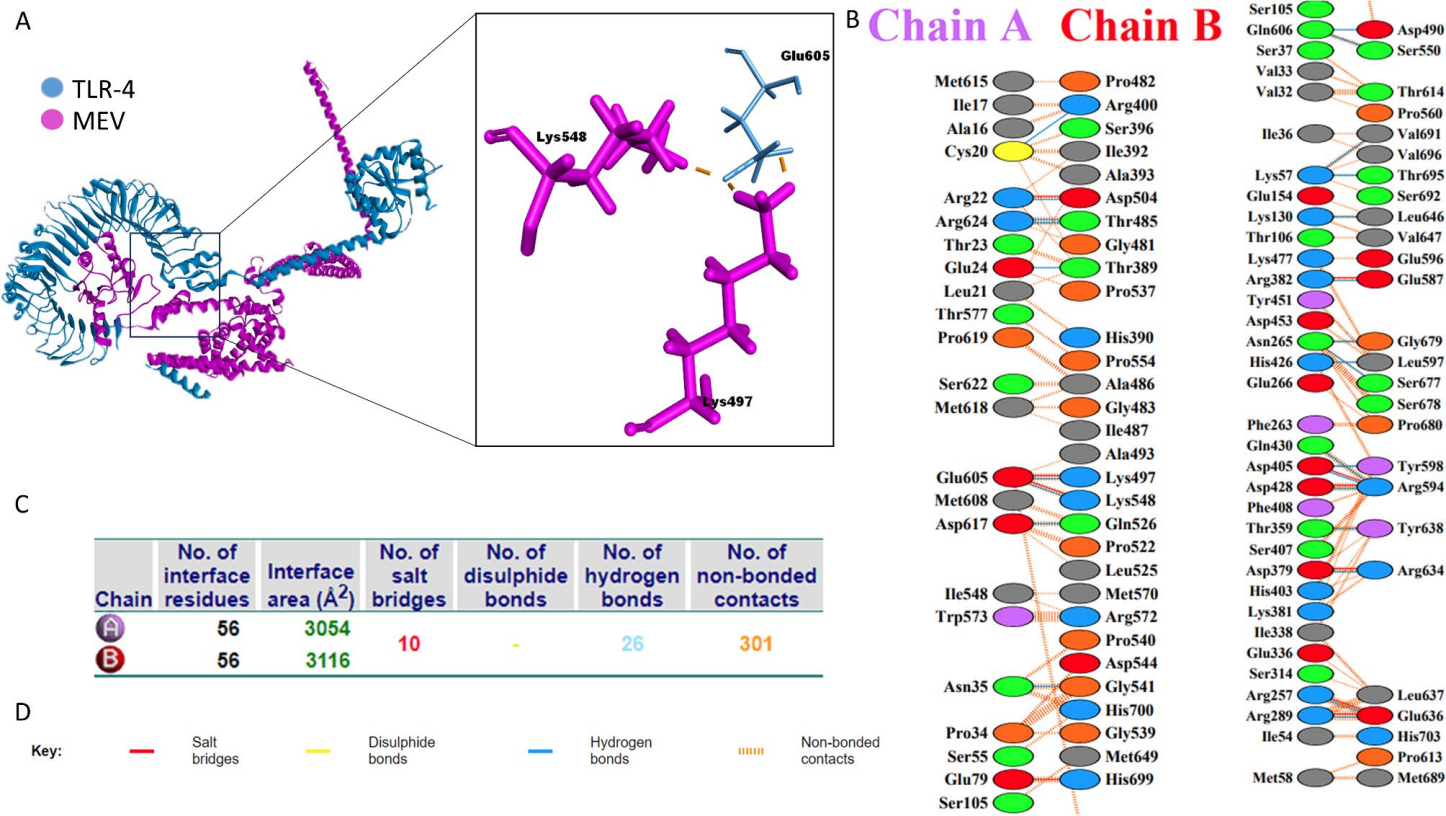
Molecular docking analysis of MEV and Bovine TLR-4. (**A**) The docked complex of bovine TLR-4 and MEV. (**B**) interacting amino acids between TLR-4 (Chain A) and MEV (Chain B). (**C**) analysis of formed bonds between docked complexes. (**D**) Key for interactions between docked molecules

**Fig. 6:**
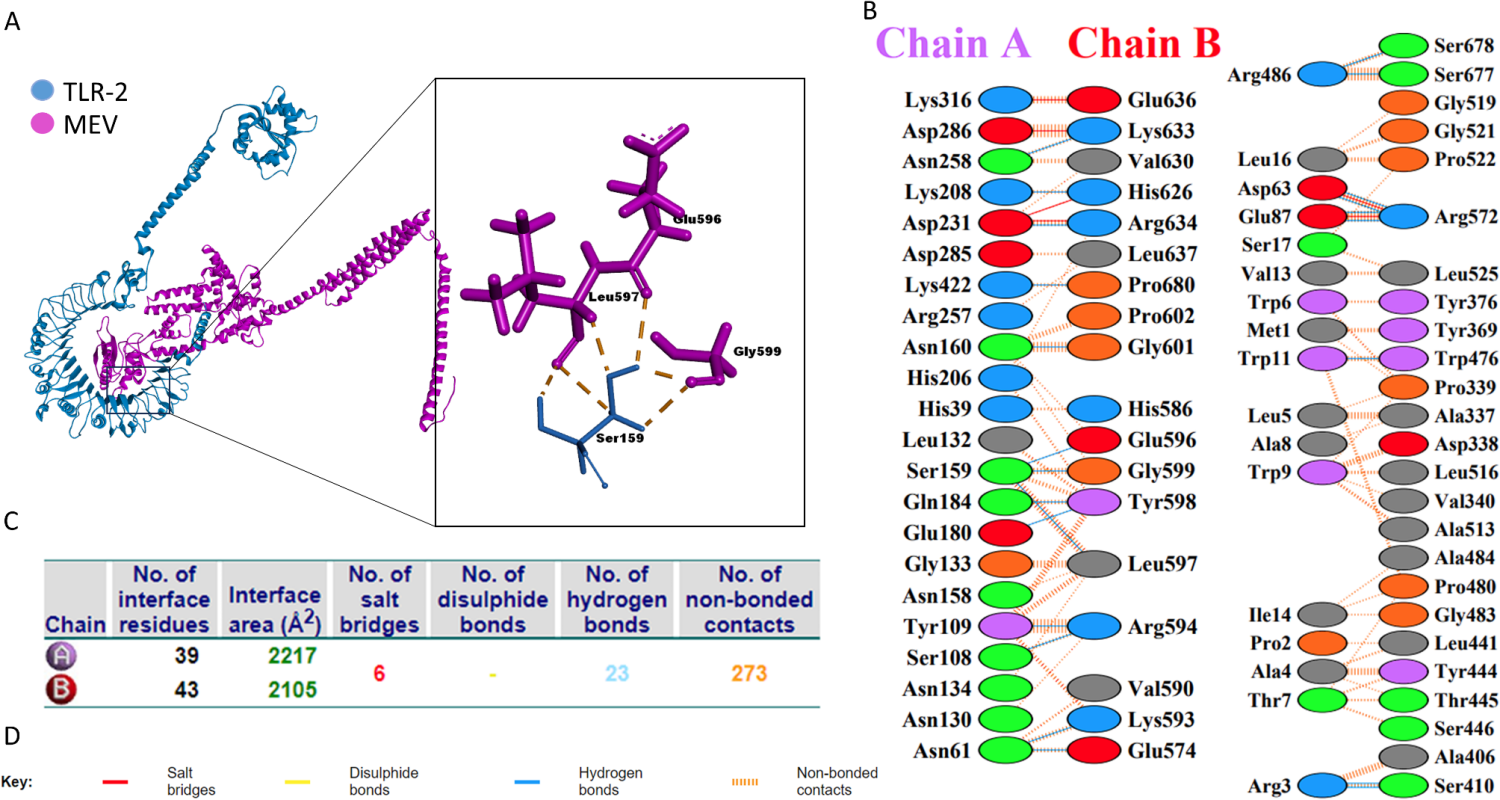
Molecular docking analysis of MEV and Bovine TLR-2. (**A**) The docked complex of bovine TLR-2 and MEV. (**B**) interacting amino acids between TLR-2 (Chain A) and MEV (Chain B). (**C**) analysis of formed bonds between docked complexes. (**D**) Key for interactions between docked molecules

The docking analysis was performed using the ClusPro web server for interaction between TLR-4 or TLR-2 and MEV. ClusPro generated 30 models for each interaction, each model was visualized and assessed based on type, location, and number of interacting atoms between the TLRs and MEV. Models that attain a high number of bonds between MEV and extracellular domains of TLRs were selected for further evaluation. Molecular docking analysis of the selected MEV and TLR-4 predicted the formation of 62 bonds with 10 salt bridges and 26 hydrogen bonds (Fig. 5). TLR-2 molecular docking analysis revealed the formation of 60 bonds that contained 6 salt bridges and 23 hydrogen bonds (Fig. 6). The selected docked models were further evaluated based on their binding affinity ($\:\varDelta\:G)$ and dissociation factor (K_d_). The final selected models for MEV-TLR-4 $\:\varDelta\:G$ value − 22 and K_d_ 2.8e-16, MEV-TLR-2 model $\:\varDelta\:G$ was − 17.8 and K_d_ 3e-13. These values indicated that selected models are energetically viable.

### Molecular dynamic simulation

**Fig. 7:**
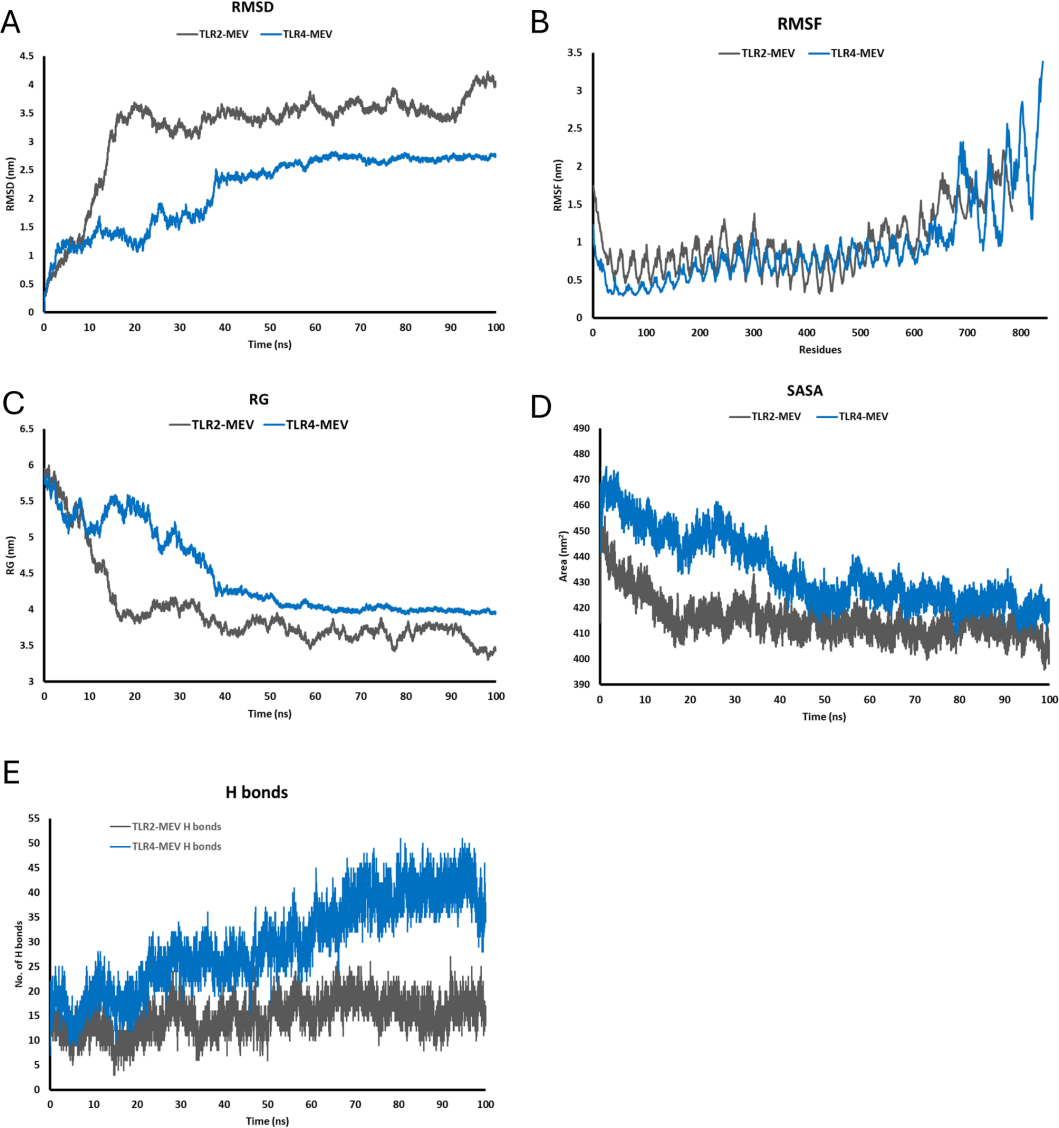
Molecular dynamic simulations graph representation of the performance of MEV construct with TLR-4/TLR-2 complexes. (**A**) RMSD plot of MEV-receptor complexes showing their stability within the 40 ns of the simulation. (**B**) RMSF plot shows good flexibility of construct with high fluctuation within the c-terminus of both receptors. (**C**) The RG plot indicates the compactness of the receptors as we progress in the simulation. (**D**) SASA plot shows higher solvent exposure of TLR-4 protein complex than TLR-2. (**E**) Hydrogen bond analysis plot showing higher number of hydrogen bond formation between MEV and TLR-4 compared to MEV and TLR-2

RMSD analysis of TLR-2 and TLR-4 protein-vaccine complexes revealed distinct patterns of structural stability throughout the simulation. Initially, TLR-4 exhibited higher RMSD than TLR-2 up to 5.5 ns (0.9110 ± 0.3025 nm vs. 0.7330 ± 0.2265 nm, respectively). However, beyond 5.5 ns, the trend reversed, with TLR2 showing higher RMSD. Both systems converged at approximately 40 ns, with TLR-4 displaying a lower RMSD compared to TLR-2 during the final phase of the simulation (40–100 ns), indicating enhanced structural stability (Fig. 7A).

RMSF analysis showed TLR-2 showed a slightly higher flexibility (0.99 ± 0.4) than TLR-4 (0.93±. 0.54). At the N-terminus, TLR-2 showed significantly greater flexibility (1.53 ± 0.14), while of TLR-4 exhibited pronounced fluctuations in the C-terminus (3.06 ± 0.23) (Fig. 7B). TLR-2 exhibited a lower RG (3.97 ± 0.56) than TLR-4 (4.47 ± 0.58), indicating a more compact structure for TLR-2. During the initial phase (up to 10 ns), both proteins showed higher RG values as they equilibrated, with TLR-2 and TLR-4 having RGs of 5.478 ± 0.28 and 5.408 ± 0.215, respectively. After 40 ns, the RG values stabilized, reflecting equilibrium conformations; TLR-2 maintained a more compact structure (3.678 ± 0.121), while TLR-4 stabilized at a higher RG (4.046 ± 0.089), with a more expanded conformation (Fig. 7C). SASA analysis showed TLR-4 had a consistently higher exposed surface area (434.43 ± 13.97) compared to TLR-2 (415.86 ± 8.03), aligned with the RG trends (Fig. 7D).

Hydrogen bonds analysis reveals distinct interaction profiles that highlight differences in binding strength and stability. On average, the interaction between TLR-4 and the vaccine formed a significantly higher number of H bonds (29.64 ± 9.00) compared to TLR-2 and the vaccine (15.01 ± 3.50). The larger but variable H bond number in the TLR4-vaccine interaction suggests a more dynamic and robust binding interface (Fig. 7E).

The MMGBSA analysis provided insights into the binding energetics of the MEV vaccine construct with TLR-2 and TLR-4. The TLR4-MEV complex exhibited a stronger total binding energy of **-226.51 kcal/mol**, mostly due the favorable van der Waals (**-304.67 kcal/mol**) and electrostatic (**-425.19 kcal/mol**) interactions, partially encountered by an unfavorable polar solvation energy (**543.15 kcal/mol**) and a stabilizing nonpolar solvation energy (**-39.8 kcal/mol**). In contrast, the TLR2-MEV complex demonstrated a weaker total binding energy of **-177.09 kcal/mol**, with a lower van der Waals (**-238.44 kcal/mol**) and electrostatic (**-234.54 kcal/mol**) interactions contributing significantly, and a lesser desolvation penalty (**326.77 kcal/mol**).

### In-silico molecular cloning

**Fig. 8:**
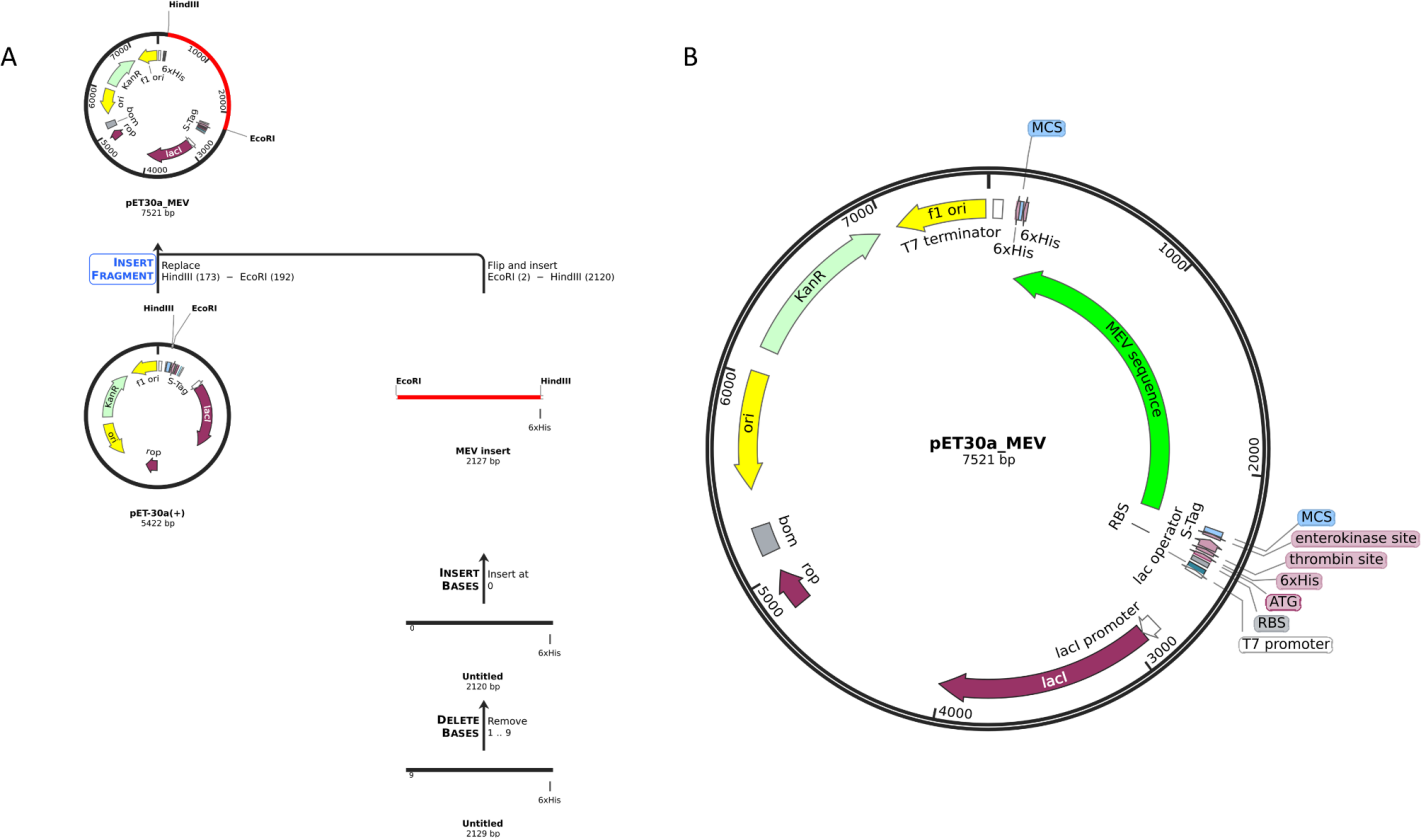
Illustration of vaccine DNA sequence cloning in pET30a(+) vector. (**A**) shows the insertion of DNA sequence in pET30 vector using EcoRI and HindIII restriction enzyme. (**B**) shows the final recombinant plasmid containing MEV sequence in green arrow

The protein sequence of MEV was reverse-translated to its respective DNA sequence using the Reverse translate tool, sequence manipulation suit. The codon optimization index was 0.91 and the GC percentage was 59.94%, indicating a good abundance of commonly used codons. (Fig. 8) shows the optimized DNA sequence ligated with the pET-30a (+) vector using EcoRI and HindIII restriction digestion.

## Discussion

Vaccination remains one of the most essential measures for the prevention and control of FMD. Traditional viral-based vaccines have great immunogenicity, but there are several drawbacks, including high manufacturing costs linked to the need for a dedicated manufacturing facility, along with the possibility of insufficient inactivation or viral leakage [55, 56]. Recombinant DNA technology provides a solution for those problems with a chance of producing a vaccine that attains long-lasting immunity. Recent publications indicated the success of using recombinant epitopes from different FMDV structural proteins to provide better viral immunity and fewer hazardous production process [57–59].

Immunoinformatics and computational biology have raised the bar in the vaccine production industry through enabling a safer and more innovative way to design viral vaccines [60, 61]. The huge amount of available biological data can be used to identify promising epitopes and help suggest vaccine candidates to be validated in wet lab [62]. Therefore, the current study aimed to develop MEV with high immunogenicity against FMDV. During this work, we identified the potential dominant B-cell and T-cell epitopes from different circulating Egyptian serotypes to help design an efficient FMDV vaccine. The suggested vaccine is predicted to provide immunity against different FMDV lineages and serotypes based on the inclusion of conserved and immunogenic epitopes. Additionally, the designed vaccine is computationally predicted to activate both humoral and cell-mediated immunity, though experimental validation is required to confirm the efficacy of these findings.

During this study, we showed the significance of employing immunoinformatics for FMDV vaccine development. Previous studies such as those by Ahmed et al. 2022 [63] and Bhutta et al. 2024 [64] utilized immunoinformatics for vaccine design focused on specific serotypes or polyprotein regions. For instance, these studies have focused only on developing FMDV vaccine against serotype O. In contrast, our study includes epitopes from multiple serotypes increasing the vaccine’s capability to provide cross-serotype immunity. Like Bhutta, we have specifically predicted epitopes for FMDV against BoLA. However, Bhutta has studied the interaction of multiepitope vaccine with specific BoLA using molecular docking. our work focused on studying the interaction of developed vaccines with different cellular receptors such as TLR-4 and TLR-2. We also focused on the use of variable structural proteins of virus capsid when other studies such as Ahmed et al. used the full polyprotein for prediction of vaccine epitopes. Using structural proteins ensures the inclusion of FMDV antigenic sites.

Several antigenic sites have been identified in highly variable structural proteins and have been divided into five main antigenic sites. Sites 1, 3, and 5 are βG-βH loop and carboxy terminus, residues 566 and 567 located in the βΒ-βC loop, and residue 672 of VP1, respectively. Antigenic site 2 is composed of residues at positions 155–158, 160,162, and 216 of VP2, whereas residues at positions 164 and 219 are responsible for interaction with monoclonal antibodies. Residues 359 and 361 of VP3 were found to be crucial for antigenic site 4 [17]. Even, VP1 is considered a highly variable protein, it contains conserved regions throughout different serotypes. These regions are crucial for cellular integration and interaction, such as G-H loop containing the “Arg-Gly-Asp, or RGD” motif [12, 65], or capsid processing such as the YCPRP sequence [66].

During this work, we predicted the linear B cell epitopes in FMDV structural proteins (VP1-3). Peptides from the VP3 protein were selected for all tested serotypes. Selected VP3 epitopes have not been experimentally discovered or identified in previous studies. VP3-131 and 132 [67] residues were predicted to have a significant antigenic property. Those residues were found within predicted B-cell epitopes with high rank and binding affinity toward B-cell receptors. However, those epitopes were tested non-antigenic by the Vaxigen webserver. A single peptide was selected from SAT2 VP2 protein that have commonly known antigenic residue in position 133 [68].

Helper T cell epitopes were identified in multiple FMDV proteins, with abundance in conserved sequences of VP4 and non-structural proteins [68]. A specific epitope in the VP4 protein was identified to have binding affinity to four different BoLA alleles [69, 70]. Being a very conserved protein among structural proteins, VP4 was beyond the scope of this study, as we were looking to enhance the FMDV vaccine with different epitopes with higher antigenicity and conservancy within FMDV isolates. A recent study was able to generate memory HTL from FMDV variable structure proteins [71]. Epitopes presented by the study were predicted in the present work, yet their binding affinity and percentile rank were lower than our selection threshold. Two epitopes, VP1(560–575) and VP1(561–576) were predicted with the required antigenicity and allergenicity from serotype A. Those epitope ranges were following experimentally identified epitopes [73]. HTL epitopes were also identified in non-structural proteins [72, 73], however, the inclusion of these epitopes into the predicted vaccine would decrease the efficiency of differentiating infected from vaccinated animals (DIVA).

For cytotoxic T-cell epitopes, fewer reports were able to identify FMDV peptides for bovine and swine MHC-1. In a study focused on identifying swine MHC-1 epitopes, authors identified high binding affinity between swine leukocyte antigen-2 and epitopes from VP1 protein [74]. Herein, we identified a similar position with high conservancy and antigenicity in the VP1 region (602–611) that has a binding affinity toward BoLA-2:00801. BoLA-2:00801 was also identified as the second most frequent MHC-1 allele in Holsten cattle [75]. Other epitopes were predicted in VP2 and VP3 proteins that have a binding affinity toward MHC-I. Such epitopes as VP3 (402–410), VP2 (256–264), and VP2 (275–283) epitopes were reported to induce T-cell proliferation [67]. The presence of different epitopes that can activate cytotoxic response indicates the need for further investigation of different structural protein (VP1-VP3) peptides in wet lab.

This study has faced two major limitations in predicting and selecting effective epitopes for FMDV. First, there are limited prediction tools that align the protein of interest against commonly identified BoLA alleles [76]. Second, since FMDV presents a high genomic variation between its serotypes and even within each serotype, this variation affects epitope selection. This variation decreased the chance of selecting common epitopes for FMDV serotypes. It is important to notice using different FMDV proteins, strains, serotypes, or updated versions of the same or another web tool may yield different epitope predictions.

HBHA was used as an adjuvant, and has been tested experimentally with MEV against FMDV. HBHA has been identified as a TLR-4 agonist with no toxicity, strong immunogenicity, and the ability to induce dendritic cell maturation [77]. HBHA would increase the chance of MEV induction of cell-mediated immunity.

Adjuvant and predicted epitopes are connected through different types of linkers. EAAAK linker forms a rigid α-helix that provides a fixed distance between different domains, hence providing good separation between them [78, 79]. EAAAK linker was used to link adjuvant and B-cell epitopes. KK linker is one of the target sequences for lysosomal protease cathepsin B that works on the antigen presentation process [80]. Besides, the KK linker increases the immunogenicity of the MEV [81]. Likewise, AAY linker can effectively separate cytotoxic T-cell epitopes, as it targets different mammalian proteasomes [82]. GPGPG linkers could induce helper T lymphocytes, a critical characteristic for MEV [81, 83]. The presence of protein linkers is important to prevent and reduce the chance of junctional immunogenicity, ensures flexible structures and low rigidity [84–86]. The final construct contained a 6x His tag to facilitate the purification of the final protein.

Constructed vaccine physicochemical properties were evaluated using the ProtParam server [87]. The vaccine’s basic nature predicted by its theoretical pI and its low GRAVY score indicates the solubility of the vaccine within the physiological environment. Previous studies such as Foroutan et al. [88] and Azami et al. [89] have used the same tools for in-silico assessment of their designed vaccine that have proved to induce a cellular and humoral response in mice through laboratory validation. The physicochemical properties predicted for the FMDV vaccine in our study were comparable with these previous studies. However, our predicted vaccines have a higher aliphatic index ensuring more thermal stability of the protein. The high pI of the FMDV ensures the presence of positively charged residues capable of interacting with negatively charged cellular receptors [90]. Toll-like receptors are recognition molecules that can be found on different cell surfaces of many cell types. Toll-like receptors contain a leucine-rich repeat domain as an extracellular domain and an intracellular toll/IL-1 receptor-like (TIR) domain. FMDV was reported to interact with different Toll-like receptors [25, 91]. For instance, TLR-3,7 and 8 were reported to interact with FMDV endosomal RNA [92, 93], on the other hand, viral VP1 and VP3 proteins interact with TLR-2 and TLR-4, respectively. The interaction between FMDV and TLR-2 induces IL-6 production, while the interaction of VP3 and TLR-4 activates the immune response toward FMDV [94, 95]. In this study, we analyzed the interaction between the designed MEV and TLR-4 and TLR-2 using molecular docking analysis. The molecular docking with TLR-4 showed many interactions between the extracellular domain of TLR-4 and MHCII and part of MHCI epitopes. In the same manner, TLR-2 showed interaction with MHCI and MHCII epitopes. The analysis of dissociation and binding affinity energies exhibited a highly stable structure between the MEV and TLRs.

Molecular dynamic simulation revealed a distinct pattern of structural stability and flexibility of binding between the MEV construct and TLR-2/TLR-4. TLR-4 RMSD analysis showed greater stability of its complex with MEV compared to TLR-2, specifically during final phase of the simulation. RG and SASA proved that TLR-4 adopts more open and exposed conformation during interaction with the vaccine, while TLR-2 maintains a more compact structure. Furthermore, hydrogen bond and MMGBSA analysis demonstrated that TLR-4-vaccine complex forms stronger and more interactions with high binding affinity (-226.51 kcal/mol) compared to TLR-2 (-177.09 kcal/mol). These results suggest that the MEV construct would have stronger and more dynamic interaction with TLR-4. This finding supports its potential role in activating TLR-4-mediated immunity.

The use of immunoinformatics and in-silico design is an important step in evaluating and predicting a new candidate vaccine, especially for highly heterogenic viruses such as FMDV. Including in-silico evaluation of vaccine candidates is essential to avoid time and financial-consuming in wet lab experimentations. Herein, we proposed a possible pipeline for designing the FMDV multiepitope vaccine. The multiepitope vaccine contained B-cell and T-cell epitopes that can induce immune system reaction in both its format, cell-mediated and humoral immunity. This approach has been applied in the design of vaccines against different pathogens such as SARS-CoV-2 [54], lumpy skin virus [25], avian leukosis virus [96], human papillomavirus [97] and *Acinetobacter baumannii* [98].

## Conclusion

The application of vaccine strategies for eradicating FMDV infections showed its effect in many countries worldwide. The process requires the application of an effective vaccine to eliminate circulating strains and prevent the introduction of new ones. In this study, we have used an immunoinformatic approach for the design and evaluation of a polyvalent multiepitope vaccine for FMDV. The introduced pipeline can be generalized to different FMDV serotypes, strains, or isolates based on circulating strains. The final construct presented in this study could protect against seven isolates of three FMDV serotypes. Multiple computational tools, including discontinuous B-cell epitope prediction, molecular docking, and molecular dynamic simulation, predicted the stability and the strong interaction of the vaccine with bovine TLR-4 and TLR-2. The physicochemical properties of the vaccine underscore its potential as a promising candidate for further experimental validation. Future work should focus on testing the proposed vaccine experimentally to ensure its protective efficacy and the capability to induce humoral and cellular immune responses.

## Electronic supplementary material

Below is the link to the electronic supplementary material.

Supplementary Material 1

## Data Availability

Additional file can be found in the Git-Hub repository through this link: https://github.com/mustafarzaher/A-novel-immunoinformatic-approach-for-design-and-evaluation-of-hepta-valent-multiepitope-FMDV.git.

## Abbreviations

FMDV: Foot-and-mouth disease virus
FMD: Foot-and-mouth disease
MEV: Multi-epitope vaccine
ML: Machine learning
NCBI: National center of biotechnology information
CTL: Cytotoxic T Lymphocytes
HTL: Helper T Lymphocytes
MHC: Major histocompatibility
IEDB: Immune epitope database
ANN: Artificial neural network
BoLA: Bovine leukocyte alleles
EA-3: Egyptian East Africa 3
TLR: Toll-like receptor
HBHA: Mycobacterium tuberculosis heparin-binding hemagglutinin
