# Supplementary material for: A novel immunoinformatic approach for design and evaluation of heptavalent multiepitope foot-and-mouth disease virus vaccine
Source: BMC Vet Res. 2025 Mar 7;21:152. doi: 10.1186/s12917-025-04509-1 (PMC11887215; doi:10.1186/s12917-025-04509-1)
Supplement: Supplementary file 1 — Supplementary Material 1 [file 12917_2025_4509_MOESM1_ESM.pdf]

Table S1 Bovine MHC-I alleles in the IEDB database that have been aligned to protein sequences.

BoLA-1:01901	BoLA-2:02601	BoLA-3:00401	BoLA-4:02401	BoLA-T2c
BoLA-1:02001	BoLA-2:02602	BoLA-3:00402	BoLA-4:02402	BoLA-T5
BoLA-1:02101	BoLA-2:02603	BoLA-3:00403	BoLA-4:06301	BoLA-T7
BoLA-1:02301	BoLA-2:03001	BoLA-3:01001	BoLA-5:00301	
BoLA-1:02801	BoLA-2:03202	BoLA-3:01101	BoLA-5:03901	
BoLA-1:02901	BoLA-2:04301	BoLA-3:01701	BoLA-5:06401	
BoLA-1:03101	BoLA-2:04401	BoLA-3:01702	BoLA-5:07201	
BoLA-1:03102	BoLA-2:04402	BoLA-3:01703	BoLA-6:01301	
BoLA-1:04201	BoLA-2:04501	BoLA-3:02701	BoLA-6:01302	
BoLA-1:04901	BoLA-2:04601	BoLA-3:02702	BoLA-6:01401	
BoLA-1:06101	BoLA-2:04701	BoLA-3:03501	BoLA-6:01402	
BoLA-1:06701	BoLA-2:04801	BoLA-3:03601	BoLA-6:01501	
BoLA-1:07401	BoLA-2:05401	BoLA-3:03701	BoLA-6:01502	
BoLA-2:00501	BoLA-2:05501	BoLA-3:03801	BoLA-6:03401	
BoLA-2:00601	BoLA-2:05601	BoLA-3:05001	BoLA-6:04001	
BoLA-2:00602	BoLA-2:05701	BoLA-3:05101	BoLA-6:04101	
BoLA-2:00801	BoLA-2:06001	BoLA-3:05201	BoLA-amani.1	
BoLA-2:00802	BoLA-2:06201	BoLA-3:05301	BoLA-AW10	
BoLA-2:01201	BoLA-2:06901	BoLA-3:05801	BoLA-D18.4	
BoLA-2:01601	BoLA-2:07001	BoLA-3:05901	BoLA-gb1.7	
BoLA-2:01602	BoLA-2:07101	BoLA-3:06501	BoLA-HD6	
BoLA-2:01801	BoLA-3:00101	BoLA-3:06601	BoLA-JSP.1	
BoLA-2:01802	BoLA-3:00102	BoLA-3:06602	BoLA-T2a	
BoLA-2:02201	BoLA-3:00103	BoLA-3:06801	BoLA-T2b	

Table S2 Bovine leukocyte alleles (i.e., BoLA-DRB3) used for prediction of MHC-II epitopes

BoLA-DRB3_0101	BoLA-DRB3_03021	BoLA-DRB3_2705	BoLA-DRB3_0701	BoLA-DRB3_3001
BoLA-DRB3_1002	BoLA-DRB3_1201	BoLA-DRB3_3401	BoLA-DRB3_1701	BoLA-DRB3_4301
BoLA-DRB3_1901	BoLA-DRB3_20011	BoLA-DRB3_5701	BoLA-DRB3_2201	BoLA-DRB3_6301
BoLA-DRB3_25011	BoLA-DRB3_2702	BoLA-DRB3_0502	BoLA-DRB3_2710	BoLA-DRB3_1001
BoLA-DRB3_3101	BoLA-DRB3_3202	BoLA-DRB3_1501	BoLA-DRB3_4001	BoLA-DRB3_1802
BoLA-DRB3_4303	BoLA-DRB3_46011	BoLA-DRB3_2004	BoLA-DRB3_6001	BoLA-DRB3_2402
BoLA-DRB3_6402	BoLA-DRB3_0303	BoLA-DRB3_2707	BoLA-DRB3_0801	BoLA-DRB3_3002
BoLA-DRB3_0201	BoLA-DRB3_1301	BoLA-DRB3_3402	BoLA-DRB3_1702	BoLA-DRB3_4302
BoLA-DRB3_1101	BoLA-DRB3_20012	BoLA-DRB3_5702	BoLA-DRB3_2202	BoLA-DRB3_6401
BoLA-DRB3_1902	BoLA-DRB3_2703	BoLA-DRB3_0503	BoLA-DRB3_2801	
BoLA-DRB3_25012	BoLA-DRB3_3203	BoLA-DRB3_1601	BoLA-DRB3_4101	
BoLA-DRB3_3103	BoLA-DRB3_4701	BoLA-DRB3_2005	BoLA-DRB3_6101	
BoLA-DRB3_4401	BoLA-DRB3_0401	BoLA-DRB3_2708	BoLA-DRB3_0901	
BoLA-DRB3_6501	BoLA-DRB3_14011	BoLA-DRB3_3501	BoLA-DRB3_1703	
BoLA-DRB3_0301	BoLA-DRB3_2002	BoLA-DRB3_5801	BoLA-DRB3_2301	
BoLA-DRB3_1103	BoLA-DRB3_2704	BoLA-DRB3_0601	BoLA-DRB3_2901	
BoLA-DRB3_1903	BoLA-DRB3_3301	BoLA-DRB3_1602	BoLA-DRB3_4201	
BoLA-DRB3_2601	BoLA-DRB3_4802	BoLA-DRB3_2101	BoLA-DRB3_6201	
BoLA-DRB3_3201	BoLA-DRB3_0501	BoLA-DRB3_2709	BoLA-DRB3_0902	
BoLA-DRB3_4501	BoLA-DRB3_14012	BoLA-DRB3_3601	BoLA-DRB3_1801	
BoLA-DRB3_6601	BoLA-DRB3_2003	BoLA-DRB3_5901	BoLA-DRB3_2401	
